# On the Optimization of a Probabilistic Data Aggregation Framework for Energy Efficiency in Wireless Sensor Networks

**DOI:** 10.3390/s150819597

**Published:** 2015-08-11

**Authors:** Stella Kafetzoglou, Giorgos Aristomenopoulos, Symeon Papavassiliou

**Affiliations:** School of Electrical and Computer Engineering, National Technical University of Athens (NTUA), 9 Iroon Polytechniou str., Zografou, 15780 Athens, Greece; E-Mails: skafetzo@netmode.ntua.gr (S.K.); aristome@netmode.ntua.gr (G.A.)

**Keywords:** wireless sensor networks, smart cities, data gathering and aggregation, network optimization

## Abstract

Among the key aspects of the Internet of Things (IoT) is the integration of heterogeneous sensors in a distributed system that performs actions on the physical world based on environmental information gathered by sensors and application-related constraints and requirements. Numerous applications of Wireless Sensor Networks (WSNs) have appeared in various fields, from environmental monitoring, to tactical fields, and healthcare at home, promising to change our quality of life and facilitating the vision of sensor network enabled smart cities. Given the enormous requirements that emerge in such a setting—both in terms of data and energy—data aggregation appears as a key element in reducing the amount of traffic in wireless sensor networks and achieving energy conservation. Probabilistic frameworks have been introduced as operational efficient and performance effective solutions for data aggregation in distributed sensor networks. In this work, we introduce an overall optimization approach that improves and complements such frameworks towards identifying the optimal probability for a node to aggregate packets as well as the optimal aggregation period that a node should wait for performing aggregation, so as to minimize the overall energy consumption, while satisfying certain imposed delay constraints. Primal dual decomposition is employed to solve the corresponding optimization problem while simulation results demonstrate the operational efficiency of the proposed approach under different traffic and topology scenarios.

## 1. Introduction

Internet of Things (IoT) is based on the vision of a worldwide network of intercommunicating devices, ranging from wearable devices such as smartwatches to sensors and mobile phones, capable of supporting the public good and leading to economic growth and personal enrichment of life. IoT is expected to connect tens of billions “things” to the Internet by the next decade, which contributes to the mashup of new synergistic services. 

Smart cities are to be created upon this technology and while several definitions exist on how a city is considered a smart one [1], all agree on the potential to improve its citizens’ quality of life. By utilizing the networked infrastructure different city’s functions can be greatly improved and aspects of social, cultural and urban development are enabled, through the use of smart devices, computers, sensors and actuators [2]. The importance of defining and creating smart cities has become a trend, yet little academic research has sparingly discussed the phenomenon. However lately, an increasing amount of initiatives can be observed such as the Array of Things (AoT) project [3] in the city of Chicago, which is built on a network of interactive, modular sensor boxes that collect real-time data on the city’s environment, infrastructure, and activity for research and public use. 

In this smart city vision, sensors and sensor networks play a vital role since they can serve as collector and/or distributor of data (e.g., related to weather conditions, traffic information *etc.*) that can contribute to the creation of enhanced services both for public and private use. Given the enormous requirements that emerge in such a setting—both in terms of data and energy—data aggregation appears as a key element in reducing the amount of traffic in wireless sensor networks and achieving energy conservation.

Data aggregation has been proposed as a data gathering paradigm in wireless sensor networks for improving energy usage efficiency and increasing the lifetime of wireless sensor networks. Data aggregation refers to any process in which information is gathered and expressed in a summary form, for purposes such as statistical analysis, and in sensor networks has been mainly implemented either by distributed source coding [4] and header compression [5], or by the utilization of some form of aggregation function in the gathered data, such as calculation of mean, maximum, minimum value *etc.* [6,7]. Data aggregation is performed either at each or at certain nodes while packets traverse the network. 

By exploiting the correlations of measurements gathered from neighboring nodes, transmission of fewer data packets with aggregated information is achieved, instead of sending individual data items from sensors to sinks. This, in turn, results in a decrease in the amount of traffic and corresponding queuing delay at each node. On the other hand, having sensors wait for data from their neighbors in order to realize aggregation, introduces some delay that adds on to the overall delay that a data packet experiences as it traverses the network.

However, though there has been observed a tradeoff between the energy savings and the delay in the delivery of the packets as data aggregation is enforced, most research efforts do not account for the aggregation time, and assume that the aggregation operation is performed on the fly. We argue that, in several cases, and mainly when significant energy savings are achieved, this time is not negligible and should be considered, especially for delay sensitive sensor applications.

Our prior work includes the introduction of a general probabilistic framework for data aggregation and processing in distributed sensor networks [8], where aggregation is performed at each node in a probabilistic and distributed manner. Specifically, each node may aggregate packets according to some pre-assigned probability for a certain pre-defined period, and after this period expires the node forwards a single aggregated data packet containing the information of all packets. Numerical evaluation results have demonstrated the significant improvements that can be achieved by such an approach both in terms of performance and operational efficiency under different settings and environments [9]. However it is noted that in that framework the values of both the aggregation probability and aggregation period at each node are pre-determined mainly through experimentation and they remain static throughout the system operation. The issue of the optimal and adaptive identification and configuration of these critical parameters, though of high research and practical importance in terms of performance improvement and operational effectiveness especially for dynamic and challenging operation environments has not yet been sufficiently addressed in the literature. 

Motivated by the above observations, the goal of this work is to introduce an overall optimization framework that identifies the optimal probability value for a node to aggregate packets as well as the optimal aggregation period that a node should wait, so as to minimize the overall energy consumption, while satisfying certain imposed delay constraints. The proposed approach is adaptive to the network traffic and therefore is suitable for dynamic operation environments and delay sensitive sensor network applications.

The rest of this paper is organized as follows. In Section 2, significant related work is presented while our work is clearly differentiated and positioned within the existing literature body. Section 3 illustrates initially the system model, and then the problem formulation along with the corresponding solution is presented. In Section 4, an algorithm is presented for minimizing the energy consumption in wireless sensor networks based on the solution provided in Section 3. In Section 5, the operational effectiveness of the proposed framework is validated and evaluated through modeling and simulation under various scenarios. Finally, Section 6 concludes the paper.

## 2. Related Work and Paper Contribution

Data aggregation is a technique that has been proposed in order to reduce the amount of data packets traversing the network thus increasing the lifetime of the sensor networks. Therefore, numerous relevant research efforts can be found in the literature that present data aggregation schemes and algorithms each one having different objectives and goals.

Heinzelman *et al.* [7] introduced an energy conserving cluster formation protocol called LEACH, which organizes sensor nodes into clusters having a designated node serve as cluster head, which among other things performs data fusion. Intanagonwiwat *et al.* [10] have developed an energy efficient data aggregation protocol, called directed diffusion, where the collection center, or any other node, requests data from the sensor nodes based on specific values. Sensor nodes having the corresponding data send them back to the requester, forming paths of information. In order to reduce the communication costs and the energy that is dissipated, data is aggregated along the way to the sink. The Cougar approach [6] treats the sensor network as a huge distributed database system where each sensor node holds part of the data. In the proposed architecture a leader node is elected where the in-network aggregation will take place or alternatively partial aggregation can be executed at the intermediate nodes to reduce the data size. A similar approach is presented by Madden *et al.* [11] where a generic aggregation service, namely the Tiny AGregation (TAG), is developed for *ad hoc* networks. Within the context of this scheme a user from outside the network poses queries to the sink which are forwarded to all nodes. Sensors that respond to the query send their data back to the sink following a routing tree rooted at the sink. As data flow to the sink it is aggregated to the intermediate nodes according to a defined aggregation function. 

Other aggregation strategies take into consideration Quality of Service (QoS) metrics while performing in-network processing. He *et al.* [12] have proposed an aggregation scheme that adaptively performs application independent data aggregation (AIDA) in a time sensitive manner. The aggregation function is performed at a completely different layer employed between the data link control layer and the network layer. Zhu *et al.* [8] presented a quality of service data aggregation and processing approach to determine whether and when to perform data aggregation. The results have shown that this method performs well both at the energy savings level as well as meeting certain QoS parameters, and therefore some of the basic principles of this approach, are adopted and extended within our framework.

However, most approaches consider data aggregation process as data traverse the network along with the routing scheme each approach uses, while most recently, researchers deal with the aggregation approach by means of optimization techniques. In [13] the authors consider the problem of optimizing the system lifetime of sensor networks in terms of the number of rounds of operation, *i.e.*, before the first network node fails. Their approach takes the data gathering operation and reduces it to a restricted flow problem with quota constraint. Based on that, they propose a polynomial time algorithm that finds an integer solution that specifies the number of data packets to be transferred between two neighboring nodes at each round. In [14] a distributed energy optimization method for target tracking applications is proposed. The sensor field is divided in clusters, following the maximum entropy clustering method, and the partial energy-efficient coverage problems are assigned to cluster heads. The cluster heads perform particle swam optimization according to some coverage and energy metrics. Moreover, a Radial Basis Function (RBF) prediction based dynamic energy management method is developed which approximates the target’s trajectory, so nodes wake up when needed, sensing accuracy is succeeded and energy consumption is minimized. In [15] the authors formulate the problem of data transport in sensor networks as an optimization problem whose objective function is to maximize the amount of information collected at sinks, subject to the flow, energy and channel bandwidth constraints. In addition, based on a Markov model, the link delay and the node capacity in both single and multi-hop environments are derived. This approach achieves high utility and low delay without congesting the network. In [16], the data aggregation scheduling problem is presented and solved. The authors base their work on maximal independent sets and present a distributed algorithm that generates a collision-free schedule for data aggregation in wireless sensor networks. The proposed algorithm operates in two phases: during phase one, a data gathering tree is produced while phase two results in the aggregation schedule. The time latency of the aggregation schedule generated by the proposed algorithm is minimized using a greedy strategy.

Most of the approaches presented above, however, do not account for the time spent for the operation of data aggregation, time which we argue plays an important role especially on delay sensitive sensor network applications. The works presented in [17,18,19] are among the very few that include in their calculations the aggregation time and attempt to determine its value. The authors in [17] consider the problem of optimal selection of aggregation nodes with time delay constraint, with the objective being the minimization of the overall energy consumption. They create a data aggregation tree and provide a mathematical model to describe the aggregation node selection problem. For the calculation of the aggregation points, the gathering tree is transformed into an equivalent binary tree and the problem is solved by dynamic programming procedure in polynomial time. In [18] two algorithms are presented which have the objective of improving the energy efficiency of the system by determining the aggregation points as well as the aggregation time, while at the same time satisfying some delay constraints. The authors adopt a localized approach that avoids the use of global information for determining the aggregation delays and therefore optimality is partially sacrificed in favor of feasibility. Moreover, a key element in this approach is the introduction of the Aggregation Gain parameter, which is a comprehensive measure of the benefits of applying aggregation to the system in terms of communication traffic reduction. Within this context, the gain has to be calculated for every possible choice of aggregation delays along the path of a message. To tackle this problem, a brute-force algorithm was presented at first; however, due to its high complexity its applicability to typical sensor nodes with low computational capabilities is quite limited. Therefore, a heuristic algorithm was presented which selects only one node for performing data aggregation, in which the whole available amount for aggregation is spent. Finally, in [19], a lifetime balanced data aggregation scheme for asynchronous and duty cycle sensor networks is presented, which dynamically adjusts the aggregation holding time between two neighboring nodes and hence balances their nodal lifetime.

Our work, though, shares similar objectives with the works presented in [17,18,19], as it significantly differs from existing literature, in that it is capable of obtaining the optimal values for the period of aggregation along with the corresponding probability of aggregation in order to achieve energy minimization. It is important to note that all the relevant works presented above, either perform aggregation at one single node or for a constant aggregation delay. The proposed framework in this paper, essentially introduces a novel optimization framework that simultaneously determines not only the nodes along the routing path of a message to perform aggregation but also the time to be spent for aggregation purposes at each node. More importantly the optimization problem is solved in a distributed fashion, by employing primal dual decomposition approach. Every node, at given time intervals, *i.e.*, at the beginning of every frame, determines the optimal values for the period and the probability of aggregation with ultimate the goal to achieve energy minimization at the network level, by utilizing only local information. The computation of the aforementioned optimal values at several time intervals in a distributed and dynamic manner ensures that the proposed approach is adaptive to the network traffic conditions, and therefore is suitable for dynamic operation environments and delay sensitive sensor network applications.

## 3. System Model and Problem Statement

### 3.1. System Model

Consider a wireless sensor network represented by a graph G=(N,L), where N is the set of |N| nodes and L is the set of |L| links. Two nodes, i,j∈N form a link (i,j)∈L, if they can communicate with each other (*i.e.*, are within communication range). The set of nodes that node i, can communicate with, form its neighborhood, denoted by N(i). Moreover, in order to collect the data from the overall network at a predetermined point/sensor, called the collection center, a data gathering tree is used, where the collection center is the root and each node belongs to the tree either as an internal node or as a leaf. According to the tree structure, each node *i* receives packets from its children nodes j∈Ch(i) in the tree, and then forwards them to its father node, forming data gathering sub-trees. Let F(i) be the list of nodes belonging to the upper layer of the same sub-tree of *i*. 

**Figure 1:**
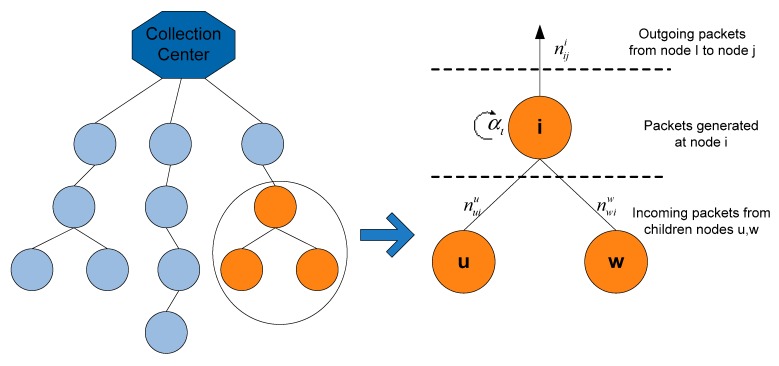
Data aggregation procedure in data gathering tree.

Every node generates packets destined to the collection center with rate $a_{i}$ where $\alpha \text{ = }(a_{1},\cdot \cdot \cdot ,a_{i},\cdot \cdot \cdot ,a_{\left|N\right|})$ denotes overall system’s generated packet rates vector. We further denote as $r_{ji}$ the incoming flow rates from all the children j∈Ch(i) of node i, and as $r_{ik}$ the corresponding outgoing flow, as depicted in Figure 1.

Moreover, each node *i* with probability $p_{i}$, where $\text{p = }(p_{1},\cdot \cdot \cdot ,p_{i},\cdot \cdot \cdot ,p_{\left|N\right|})$, enters a waiting stage of period $\tau_{i}$ during which all incoming or newly created packets are queued, while after the period expires the node forms and sends a single aggregated data packet containing the information of all packets in its queue. Otherwise, with probability $1-p_{i}$, the node sends the first packet in his queue without performing any aggregation. Each node decides whether or not to perform aggregation in a distributed manner, exploiting only locally available information. 

Each node is assumed to consume energy each time a packet is sent or received ($E_{tr}$ and $E_{rec}$ respectively). Therefore, following [7], we have:

$$E_{rec}=e_{elec}k,\text{ and }E_{tr}=e_{elec}k+e_{amp}kd^{2}$$

where, $e_{elec}$ denotes the energy that the radio dissipates to run the transmitter or receiver circuit, $e_{amp}$ the energy consumed by the transmitter amplifier, k equals to the data packet size in bits and d represents the distance in meters of the communication pair.

### 3.2. Problem Statement and Formulation

In accordance with the previous system model and considering an aggregation period, the total cumulatively energy consumed by a node for a given timeframe T is modeled as:

(1) $$E(k,d,p_{i},n)=e_{elec}k\sum_{u\in Ch(i)}n_{ui}^{u}+p_{i}(e_{elec}k+e_{amp}kd^{2})+(1-p_{i})(e_{elec}n_{ij}^{i}+e_{amp}n_{ij}^{i}kd^{2})\;$$

It is noted, the $E_{rec}$ for each node depends on the number of the data packets $\sum_{u\in Ch(i)}n_{ui}^{u}$ received from all its children, whereas the $E_{tr}$ depends on the number of packets sent, which in turn depend on the aggregation probability, *i.e.*, with probability $p_{i}$ the node enters the aggregation mode and after the aggregation period expires sends only one packet with *k* bits, and with probability $1-p_{i}$ sends $n_{ij}^{i}$ separate packets for a total of $n_{ij}^{i}k$ bits of information.

Considering a fixed packet size *k* and given a known topology, where the distances *d* between neighboring nodes, as well as the number of incoming and outgoing packets are known, the energy function $E(k,d,n,p_{i})$ depends on the aggregation probability $p_{i}$. Thus, $E(k,d,n,p_{i})\equiv E(p_{i})$ is a strictly concave, decreasing and twice continuously differentiable in the interval [0,1] function of the probability aggregation variable $p_{i}$ ($0\leq p_{i}\leq 1$).

Moreover, we assume that each packet is associated with a delay constraint representing its remaining lifetime to arrive to the collection center before expiring. The delay constraint $D_{c}$ of a packet depends on the QoS requirements posed by the application, is assumed to have an initial value, and is reduced as the packet stays in the network. The delay constraint is an application specific parameter, and thus each corresponding application can set its own value. This constraint is simply taken as an input parameter to our proposed framework, and should be propagated to the sensor network through the sink gateway, so that it can be considered and respected throughout the operation of the algorithm. Though the determination of the actual value of this parameter is not the main focus of the paper, it is expected that the delay QoS metric relates more to monitoring applications, such as indoor living monitoring [20,21] and event detection and reporting [22], as well as other delay sensitive applications such as emergency response [23], plant automation and control [24], healthcare [25], *etc.*

Our main objective is to minimize the overall energy consumption of the network towards increasing its lifetime, while satisfying packets’ Quality of Service prerequisites posed by the initial delay constraint D. Thus, the optimal aggregation probability $p_{i}$ along with the corresponding optimal aggregation period $\tau_{i}$, for every node i∈N, that minimizes the total energy consumption in the network while satisfying the packet delay constraints have to be determined.

In line with the previous analysis the Global Problem (**GP**) is defined as:

(2) $$\min_{\vec{p}}\sum_{i}^{N}E_{i}$$

(3) $$st.{\text{ e2e}}_{n^{i}}\leq D_{c}$$

(4) $$0\leq p_{i}\leq 1$$

(5) $$\tau_{i}\geq 0$$

where $e2e_{n^{i}}$ corresponds to the total delay that a packet $n^{i}$ experiences from its generation at node i to its arrival at the collection center, and comprises of (a) the time spent at node *i* (aggregation, service and queuing -$Q_{i}$- time; (b) the time needed for the transmission of a packet to the collection center, which depends on the position of node *i* to the data gathering tree, as well as (c) the time spent at the upper layer nodes of node i,j∈F(i). $D_{c}$ refers to the delay constraint, that is the time within which each packet should arrive at the collection center before expiring. Therefore, each packet has to satisfy Constraint (3).

### 3.3. Assumptions and Justification

**AS1:** Problem (1) is strictly feasible (Slatter condition qualification [26]), that is there always exists a vector $\underset{p}{\to }$ that Constraints (2) and (3) strictly hold.

**AS2:** We assume that time is divided into frames and each frame into T timeslots. Nodes can send and receive only one packet of fixed size k in each timeslot. At the beginning of each frame, problem GP is solved deriving the probability $p_{i}$ for performing aggregation and the corresponding $\tau_{i}$ aggregation period for the aggregation process. 

**AS3:** Given the difficulty in the calculation of the end-to-end delay for a packet *n^i^* prior to its arrival to the collection center, we adopt the following approximation approach. Each node i periodically informs its children nodes u∈Ch(i) of the average queuing and processing delay, as well as the values of $p_{i}$, $\tau_{i}$ observed during the previous timeframe. Then each node u∈Ch(i) adds its own average delay with its father and informs its corresponding children. This way, exploiting only locally available information, each node, independently of its location in the data gathering tree is able to estimate the queuing and processing delay that will be incurred from the upper layer nodes. Moreover, in a similar fashion, using past information each node is able to estimate the number of packets that is expected to receive from its children nodes. More specifically, at the beginning of each timeframe, nodes receive values $p_{i}$ and $\tau_{i}$ from their neighbors, while using exponential averaging techniques the long term behavior of their neighbors can be calculated. We argue that this kind of information can be easily incorporated in existing signaling.

**AS4:** During the aggregation process, a single packet is formed containing the aggregated measurement information of all packets in the queue. The aggregation function performed (e.g., min, max, average, *etc.*) depends on the application. Moreover, the aggregated packet is assumed to have the same size k as any other measurement data packet.

**AS5:** We consider an ideal Medium Access Control (MAC) layer, *i.e.*, we do not account for retransmissions and dropped packets due to collisions. A packet delivery is considered successful if it arrives at the collection center within D, otherwise it is dropped.

Based on AS2 and AS3, we can further analyze Constraint (3) as follows:

(6) $$p_{i}\tau_{i}+(1-p_{i})\min\left[Q_{i},T\right]+t_{tr}+\sum_{j\in F(i)}(p_{j}\tau_{j}+(1-p_{j})\min\left[Q_{j},T\right])\leq D_{c}$$

In accordance to this a data packet, arriving or generated at a node, experiences in the worst case the following delay: With probability $p_{i}$ waits for time $\tau_{i}$, *i.e.*, for the aggregation period to expire, while with probability $1-p_{i}$ waits for its turn to be served from the queue without performing aggregation. We argue that this time is the minimum between the length of the queue $Q_{i}$ and the timeslots T that constitute a frame. Note that $t_{tr}$ corresponds to the time needed for the transmission of a data packet from node *i* to the collection center, and it depends on the position of the node in the data gathering tree. The rest of the left hand side of Equation (6) corresponds to the delay a packet will sustain on its way to the collection center, emanating from the behavior and status of the upper layer nodes (*i.e.*, aggregation or not) in the same sub-tree in line with AS3.

### 3.4. Primal Dual Decomposition on GP

The convex optimization problem GP has two features which facilitate a distributed solution. First, objective (2) is a sum of |N| energy cost functions $E_{i}(p_{i})$ that depend only on $p_{i}$, pertaining one for each sensor node *i*. Moreover, the domain of GP enjoys the property of natural decomposing into a Cartesian product, *i.e.*, p=[0,...1]×⋅⋅⋅×[0,...1]. 

Based on the latter features, we devise a Lagrangian-based algorithm that obtains the solution of problem GP (*i.e.*, deriving optimal values of $p^{*}$ and $\tau^{*}$ for each node i).

Applying primal-dual decomposition [27] on GP, results in:

- N independent Sensor sub-Problems (SP). Considering first primal decomposition of GP by fixing τ, GP breaks into N independent sensor sub-problems, each one responsible for computing the optimal aggregation probability $p_{i}^{*}$ for a given aggregation period.
- A Master Problem (MP), responsible for updating the value of τ towards obtaining $\tau^{*}$. To solve (MP), we use a subgradient method exploiting the information of SPs’ Lagrange multipliers $\lambda_{i}^{*},i\in \;|N|$ associated to the Constraint (3).

It is important to note, that since GP is a convex optimization problem, both the master problem (MP) and sub-problems (SP) are also convex optimization problems [28]. In the following, we provide the solutions of SP and MP.

(7) $$\mathrm{SP:}\;\min E_{i}(p_{i}\text{)}$$

(7a) $$p_{i}\tau_{i}+(1-p_{i})\min\left[Q_{i},T\right]+t_{tr}+\sum_{j\in F(i)}(p_{j}\tau_{j}+(1-p_{j})\min\left[Q_{j},T\right])\leq D_{c}$$

(7b) $$0\leq p_{i}\leq 1$$

Given the monotonically decreasing nature of energy cost function $E_{i}(p_{i})$, combining Constraints (7a) and (7b), and after some mathematical manipulation, for a given $\tau_{i}$ we have:

(8) $$\begin{array}{l} p_{i}=\left\{ \begin{array}{ll} \min\left[\frac{D_{c}-\min[Q_{i},T]-t_{tr}-\sum_{j\in F(i)}p_{j}\tau_{j}+(1-p_{j})\min[Q_{j},T]}{\tau_{i}-\min[Q_{i},T]},1\right] & ,\text{for }\tau_{i}\geq \min[Q_{i},T]\\ 1 & ,\text{for }\tau_{i}\leq \min[Q_{i},T] \end{array}\right. \end{array}$$

Let $\lambda_{i}$ (or $\lambda =(\lambda_{1},...,\lambda_{N})$ and be the Lagrange multiplier corresponding to the relaxation of Constraints (8). The Lagrangian of (SP) is then given by:

(9) $$L(\vec{p,}\vec{\lambda })=E(p_{i})-\lambda \left[\min\left[\frac{D_{c}-\min[Q_{i},T]-t_{tr}-\sum_{j\in F(i)}p_{j}\tau_{j}+(1-p_{i})\min[Q_{j},T]}{\tau_{i}-\min[Q_{i},T]},1\right]-p_{i}\right]$$

The dual function and problem are respectively (Λ collectively denotes all multipliers):

(10) $$g(\Lambda ):=\underset{\left\{p_{i}\right\}}{\min}L(\Lambda )$$

(11) $$\underset{\Lambda \geq 0}{\min}g(\Lambda )$$

(11a) $$\text{st. }\lambda_{i}\geq 0$$

Taking into consideration **AS1** as well as the convexity of the primal and the dual problems, strong duality holds (duality gap is zero) and therefore solving the dual problem can equivalently solve the primal. Furthermore, taking into account the second order necessary conditions for optimality, *i.e.*, $\nabla_{p}L(p,\Lambda ){|}_{\begin{array}{l} p=p^{*}\\ \lambda =\lambda^{*} \end{array}}=0$, after some manipulation we get:

(12) $$\lambda_{i}^{*}=E_{tr/rec}\left(\min\left[Q_{i},T\right]-\left\lceil \frac{T}{\tau_{i}}\right\rceil \right)$$

Concluding the above analysis, the SP algorithm for sensor *i*, takes as input any τ and obtains the corresponding $p_{i}^{*}$ as well as the optimal value of $E_{i}^{*}(p)$.

In line with the previous analysis, given the optimal aggregation probability vector $\vec{p_{i}^{*}}$, ∀i∈|N| , the Master Problem (MP) can be defined as:

(13) $$\underset{\vec{p}}{\min}\sum_{i}^{N}E_{i}^{*}(p\text{)}$$

$$\text{st. }\tau_{i}\geq 0$$

Towards solving MP, we apply a subgradient method [27], in which it has been shown that in primal dual decomposition the subgradient of each $E_{i}^{*}$ is equal to the optimal Lagrange multiplier corresponding to the Constraint (7a) in SP. Therefore $\nabla E_{i}^{*}(p)=\lambda_{i}^{*}(p_{i})$. Finally, the global subgradient is

$$\nabla_{p}E_{i}^{*}(p)=\sum_{i\in N}\lambda_{i}^{*}(p_{i})$$

Thus, the **MP** can be solved using the following subgradient method via updating the aggregation period *τ_i_*, as follows:

$$\left[\begin{array}{l} \tau_{1}(t+1)\\ \;\vdots \\ \tau_{\text{N}}(t+1) \end{array}\right]=\left[\begin{array}{l} \tau_{1}(t)\\ \;\vdots \\ \tau_{\text{N}}(t) \end{array}\right]\pm \varepsilon (t)\left[\begin{array}{l} \lambda_{1}^{*}(p_{1}(t))\\ \;\vdots \\ \lambda_{N}^{*}(p_{N}(t)) \end{array}\right]$$

where *t* denotes algorithm’s iteration and ε(*t*) is the subgradient step.

Therefore, the pre-described subgradient update can be performed independently by each sensor node *i*, simply with the knowledge of its correspondent (**SP**) problem Lagrange multiplier $\lambda_{i}^{*}$, which in turn is also independently computed and updated by each node’s *i* (SP_i_). 

### 3.5. Convergence and Optimality

The selected step for the proposed subgradient method is a diminishing step size rule ε(*t*), with the following properties: ε(t)≥0, $\underset{t\to \infty }{\lim}\varepsilon (t)=0$ and $\sum_{t}^{\infty }\varepsilon (t)=\infty$. In accordance to [26], using a diminishing step size rule, for example $\varepsilon (t)=\frac{t}{\beta +t}$ where β>1 is a fixed constant, the subgradient algorithm converges, *i.e.*, $p(t)\to p^{*}$ as t→∞.

## 4. Aggregation-Based Energy Management (AEM)

In this section, following the reasoning and analysis presented above, a distributed algorithm, namely Aggregation-based Energy Management (AEM), is presented and applied to a wireless sensor network with the purpose of minimizing the overall energy consumption. Specifically, we first illustrate the algorithmic steps of the proposed optimization framework and then discuss how it can be applied to a wireless sensor network operating in a distributive fashion.

As already stated, the algorithm assumes the existence of a routing scheme/structure, preferably a tree based, for the collection of the sensor data from the overall network at a predetermined point/sensor, called the collection center. The first time of its operation, initialization takes place where each node initializes the aggregation probability $p_{i}$ to any value in the range of , and the aggregation period equal to $\tau_{i}=T$, where T the number of timeslots in the frame. For the subsequent operation of the algorithm, at the beginning of each timeframe every node i∈N receives from its children u∈Ch(i) and from its father j∈F(i) the corresponding aggregation probabilities as well as the aggregation periods for the previous timeframe. These values are used for the calculation of the expected delay that a packet is most likely to experience in the upper layer of the data gathering tree, in accordance with Relation (8). Moreover, each node *i* solves the (SP) problem and obtains the $p_{i}^{*}(t)$ and the Lagrange multiplier $\lambda_{i}^{*}(p_{i}(t))$ for a given $\tau_{i}(t)$. After that, the MP problem is solved, where with knowledge of the Lagrange multipliers the value of ${\text{τ}}_{i}(t+1)$ is retrieved and used again in the SP. The algorithm iterates until it converges to the optimal values of $p^{*}$ and $\tau^{*}$. Table 1 illustrates the steps of the AEM along with the expected inputs and outputs.

**Figure 2:**
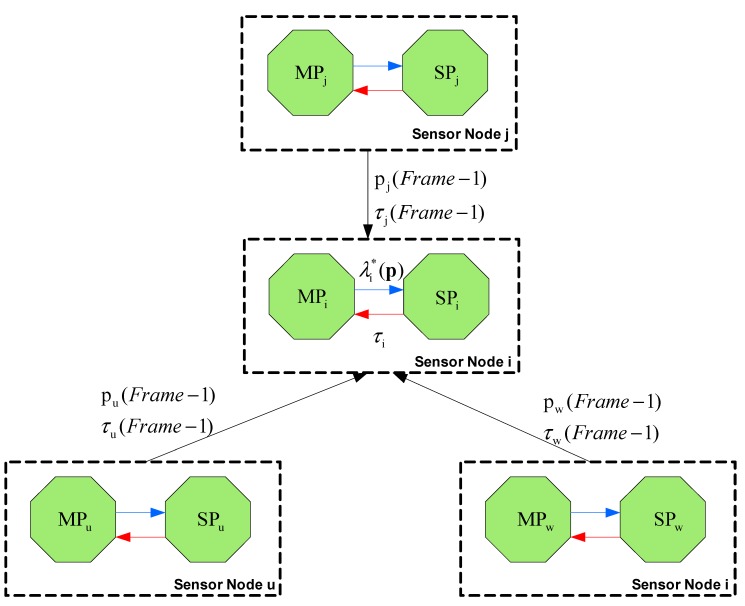
Schematic representation of the AEM algorithm.

It should be noted that, if the delay constraint cannot be met, there is no feasible solution and therefore the packets cannot be delivered to the destination within the required time constraint. Therefore, in cases where at some nodes the delay constraint cannot be satisfied during one or multiple frames, the data packets generated during that period are dropped to avoid further congesting the network. A schematic representation of the AEM algorithm is illustrated in Figure 2.

**Table 1:** Operation of aggregation-based energy management (AEM) algorithm.

Initialization	For Frame=0, $\tau_{i}=T$ and $p_{i}\;\in \left[0,1\right]$		
***Step***	*Operation*	*Input*	*Output*
**1**	Transmission to every node i ∈N of $p_{u}\left(Frame-1\right),\;\tau_{u}\left(Frame-1\right),\;\;$*u* ∈ *Ch*(*i*), and $p_{j}\left(Frame-1\right),\;\tau_{j}\left(Frame-1\right),\;j\in F\left(i\right)$		
**2**	Solve SP for every node in accordance with Section 3.4, determining the optimal aggregation probability $p_{i}^{*}\left(t\right)$ and the Lagrange multiplier $\lambda_{i}^{*}\left(p\left(t\right)\right)$, for given $\tau_{i}\left(t\right)$	$D_{C}$ $\tau_{i}\left(t\right)$	$p_{i}^{*}\left(t\right)$ $\lambda_{i}^{*}\left(p\left(t\right)\right)$
**3**	Solve the Master Problem (**MP**) for minimizing the system energy consumption and update $\tau_{i}\left(t+1\right)$	$p_{i}^{*}\left(t\right)$ $\lambda_{i}^{*}\left(p\left(t\right)\right)$	$\tau_{i}\left(t+1\right)$
**4**	Set *t* ← *t* + 1 and send *τ_i_* (*t* + 1). If termination condition does not hold go to step 2. Else algorithm has converged and optimal p^*^ and τ^*^ values have been determined.		(p^*^, τ^*^)

## 5. Evaluation Results

In this section, we will comprehensively evaluate the integrated performance of the proposed AEM algorithm through modeling and simulation, on a uniform distributed deployed network with general parameter configuration. 

### 5.1. Simulation Setup

The MATLAB tool was used for conducting simulations in a uniform distributed network area of size 200 m × 200 m. comprising of 50 nodes. The collection center is situated at the lower left area and constitutes the root of the created routing tree, which is created by use of the HELLO algorithm as described in [9].The transmission range of nodes is set to 48 m and nodes have known locations and distances one from another 20–30 m. Nodes consume energy each time the send and/or receive data packets according to the model presented in [7], as well as a constant amount of energy each time they produce a sensing measurement from the environment. Each packet, either single or aggregated, has size of 100 bytes. Moreover, the length of the frame *T* is 20 timeslots each one with duration 1 s. Overall, for each simulation 50 independent experiments were conducted for 1000 frames. It is noted that we have also experimented with larger frame sizes, such as ones comprising of 100 timeslots, where it was discovered that the size of the frame does not affect the operation of the proposed framework, either in terms of energy consumption or the probability of successful delivery. Finally, nodes store information related to the aggregation probability and period for their neighbors for the last frame and an exponential averaging function $S_{t}=aY(t-1)+(1-a)S_{t-1}$ is used for the calculation of current values as required by AS3, where constant *a* is set to 0.5.

### 5.2. Simulation Results

The key metrics used for evaluation are the probability of successful delivery of packets at the collection center (*i.e.*, within the time delay constraint), the overall energy consumption, as well as the information gain. The proposed algorithm is initially compared against (a) operation without data aggregation (*i.e.*, $p_{i}$ = 0); and (b) operation scenarios with constant probability of aggregation (*i.e.*, $p_{i}$= 1 and $p_{i}$ = 0.5) and scenarios with fixed aggregation period (*i.e.*, $\tau_{i}$ = 5 s, $\tau_{i}$ = 10 s, $\tau_{i}$ = 15 s, and $\tau_{i}$ = 20 s). Please note that the selected scenarios with their corresponding parameters are generic enough to account for different data aggregation approaches that can be found in the literature, representing several generic data gathering algorithms that either perform aggregation as data traverse the network or just forward the data to their next hop neighbor. Simulations were conducted for three types of traffic (light—2 pkts/frame, medium—4 pkts/frame and high—10 pkts/frame) and for two delay constraints *D*, one strict—20 s and one more conservative 40 s. 

In addition, in order to provide a comprehensive and comparative evaluation of our proposed framework for benchmarking mainly purposes, we have further compared AEM with: (a) the algorithm presented in [18] which determines the aggregation points and corresponding aggregation delays and (b) a basic algorithm [29] that uses an equal distribution of the maximum allowed delay among all nodes on the path. The selected evaluation metric is the total aggregation gain—explained in detail later in the paper in Section 5.2.4—which measures the benefits of aggregation in terms of communication traffic reduction. For fairness purposes, all compared algorithms use the same data routing tree for the collection of data to the collection centers. 

#### 5.2.1. Probability of Successful Delivery

**Figure 3:**
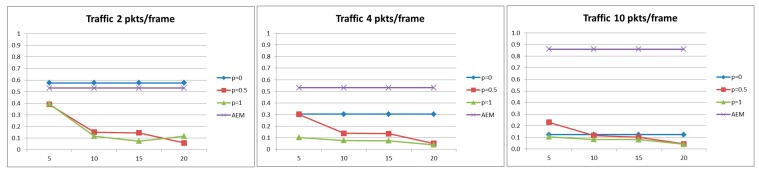
Probability of successful delivery for *D* = 20 s.

**Figure 4:**
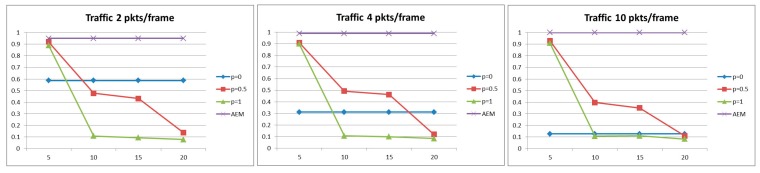
Probability of successful delivery for *D* = 40 s.

As can been observed in Figure 3, for the case of light traffic and for strict delay constraint, the case where no aggregation is performed exhibits better performance compared with the algorithms where aggregation is performed. This is due to the fact that under light load, a packet may miss the delay constraint due to the introduction of the deferred period for aggregation, since some packets that otherwise could have been transmitted may have to wait for the aggregation. However, as the traffic load increases, in a system without data aggregation, the network becomes congested and the queuing time at each node becomes the dominant factor. On the other hand, performing data aggregation implicitly results in a reduction of the network traffic load. While conducting the series of simulations, it was observed that, for high traffic load, the AEM algorithm decides for more aggregations to occur (higher values of aggregation probability) but for smaller aggregation periods . Moreover, as illustrated in Figure 4, the proposed AEM algorithm outperforms the other approaches for a less strict delay constraint. Finally, it is worth noticing that the approaches where aggregation is performed with probabilities $p_{i}$ = 1 and $p_{i}$ = 0.5 and with constant aggregation periods $\tau_{i}$, result in low probability of successful delivery, especially for strict delay constraint.

#### 5.2.2. Energy Consumption

**Figure 5:**
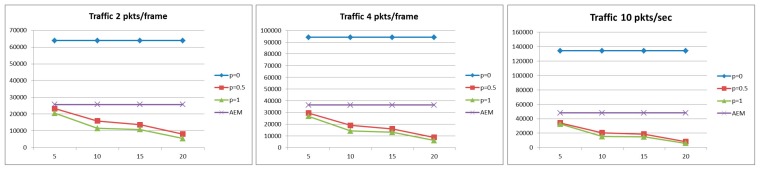
Energy Consumption for *D* = 20 s.

**Figure 6:**
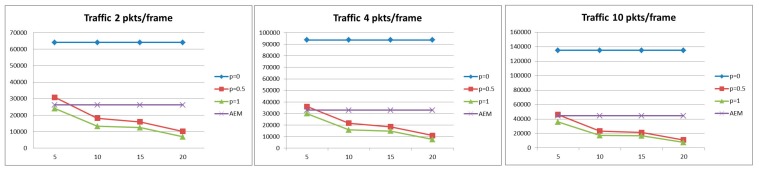
Energy Consumption for *D* = 40 s.

Figure 5 and Figure 6 illustrate the energy consumption for all compared methods under different traffic loads and delay constraints. As expected, the case where aggregation occurs is more energy efficient since less data is transmitted, and in particular the higher the probability of aggregation the less the energy consumption. However, as it was observed in the previous section, the methods with constant aggregation probability and period demonstrate low probability of successful delivery, and therefore the low power consumption is also attributed to the small amount of packet transmissions. In order to properly evaluate our AEM algorithm as far the minimization of energy consumption is concerned, we compare AEM with the method where aggregation period is set to τ = 5 s and the delay constraint is set to *D* = 40 s, Figure 6, where we observe that AEM consumes 10% less energy.

#### 5.2.3. Information Gain

Another interesting aspect of the data aggregation is the loss of information that occurred when concatenating data packets. Therefore, we define the Information Gain, $Info_{gain}$, metric, where we calculate the quality of information, *i.e.*, the percentage of initial information that is finally received at the collection center. Information gain is defined as: $Info_{gain}=1-Info_{loss}/Pkts\_arrivals$, and the value of $Info_{loss}$ is defined as:

(14) $$Info_{loss}=\sum_{i=1}^{\left|levels\right|}aggr.\;\cos t\;function(i)*Pkts\;aggregated\;i\;times$$

More specifically, we consider that each time aggregation is performed in a data packet, the information conveyed in the packet experiences a loss in accordance with the aggr. cost function(i) function. We argue that each data packet will at most be aggregated as many times as the levels of the data gathering tree, *i.e.*, the distance in number of hops from the node that the packet originates until it reaches the collection center. At the collection center, we can infer the number of aggregations that a packet has experienced as it traverses the network (Pkts aggregated i times). Information loss is also affected by the aggregation function that is used, *i.e.*, different losses occur by averaging the values rather than keeping the largest one. For the sake of simplicity, we have used the following aggregation function: aggr. cost function(i)=(10−i)/10, which for i=2, *i.e.*, a packet has been aggregated two times, the data loss is 20%. Though the used function is rather a simplistic and somewhat strict one, more complex functions can be used to simulate different aggregation types.

**Figure 7:**
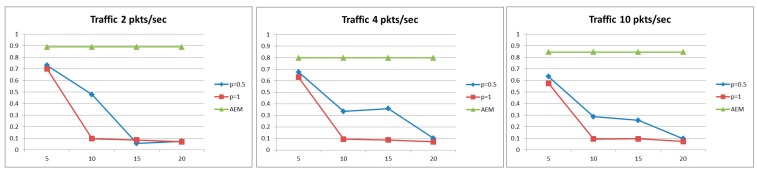
Information gain for *D* = 40 s.

Figure 7 presents the Information gain for the AEM algorithm compared against methods that perform aggregation with a constant value ($p_{i}=1$ and $p_{i}=0.5$) for delay constraint *D* = 40 s. As it can been observed, the higher the aggregation period, the less the Information gain, due to the fact that more packets are aggregated. The AEM algorithm succeeds in increasing the accuracy of the transmitted data, since it does not perform aggregation at every node as a packet traverses the network but selects the optimal ones. It is interesting to notice that most aggregations occur at the upper levels of the data gathering tree where congestion is usually more intense.

#### 5.2.4. Total Aggregation Gain

For benchmarking purposes and in order to provide a comprehensive comparative evaluation of our proposed framework which better helps positioning our framework within the existing literature, further simulation experiments were conducted that compare the proposed AEM algorithm with two different algorithms that primarily share the same objective, *i.e.*, minimize the total energy consumption. In the following, we refer to the first algorithm as Algorithm 1 presented in [18] and to the second one as Algorithm 2 presented in [29], taking into consideration the time spent at nodes for the aggregation operation and properly determining the aggregation nodes. In particular, Algorithm 1 [18], which has already been thoroughly discussed in Section 2, determines the aggregation points and corresponding aggregation delays, while Algorithm 2 adopts an equal distribution of the maximum allowed delay among all nodes on the path. Algorithm 2 is based on a basic aggregation algorithm [29] that performs data-centric routing. The routing scheme used is the data gathering tree constructed at the beginning of the network operation and aggregation is performed at every node for a constant amount of time, *i.e.*, equally distributing the allowed aggregation delay among all the nodes in the path. The selected evaluation metric for this comparative evaluation is the total aggregation gain which measures the benefits of aggregation in terms of communication traffic reduction. The aggregation gain G is calculated based on the following equation (as defined in [18]): $G=1-\frac{t_{a}}{t_{o}}$, where $t_{a}$ corresponds to the number of transmissions when aggregation is applied while $t_{o}$ accounts for the number of transmissions for the data gathering to be performed (without applying any aggregation approach). The maximum allowed aggregation delay is set to 20 s for all approaches, and different packet generation rates were considered thus producing a varying number of packets during the corresponding interval. The simulation setup that was used is the same one described in Section 5.1.

**Figure 8:**
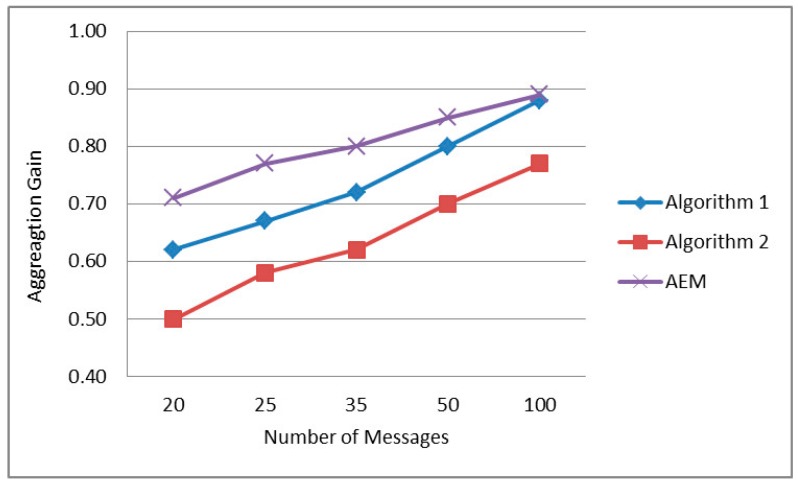
Total aggregation gain as a function of network traffic.

As observed by the results presented in Figure 8, our approach outperforms both compared algorithms by showing a great increase with reference to the aggregation gain. It can be seen that as the traffic in the network increases, the Aggregation Gain also increases for all algorithms, confirming the benefits of aggregation with respect to the decrease in the amount of messages in the network, especially under heavy traffic scenarios. Furthermore, it is clearly demonstrated that the proposed AEM algorithm significantly outperforms both other considered algorithms for all simulated scenarios This is accounted for the fact that AEM neither spends all the aggregation delay in one node (as Algorithm 1 does), nor distributes the allowed aggregation delay equally to every node (as Algorithm 2 does). Instead, through the proposed optimization framework, it selects different nodes with optimal aggregation delays resulting in performing more efficient aggregation decisions resulting in fewer transmissions.

#### 5.2.5. Capability to Adapt in Changing Conditions

Finally, the capability of the proposed framework to changes in the traffic load and the application requirements is also evaluated. To achieve this, the following scenario is considered. A sensor network, consisting of fifty randomly deployed environmental sensor nodes in an area of 50 m × 50 m, is used to produce measurements for an air pollution application. Under this scenario, sensor nodes generate packets, with air quality readings, at a rate of 0.2 packets per second with time delay constraint of 40 s. After a period of 50 time frames, an elevated value of the measured element is reported triggering a “high” air pollution event, and therefore it needs to be reported at the collection center within a strict delay constraint, *i.e.*, 20 s, while at the same time, nodes generate packets at a quicker rate (*i.e.*, 0.5 packets per second). When the event expires (e.g., after 50 frames), the sensor network continues its normal operation. 

As before, our AEM algorithm is compared with several other operation scenarios of constant aggregation probability and scenarios with fixed aggregation periods.

**Figure 9:**
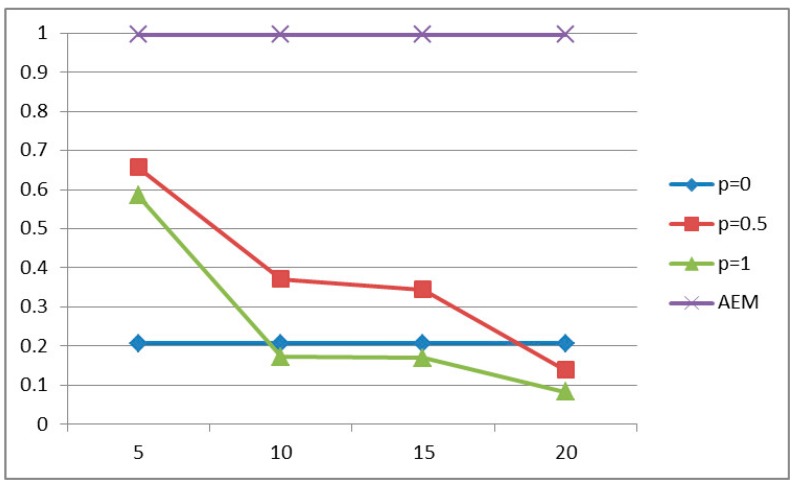
Probability of successful delivery of packets.

**Figure 10:**
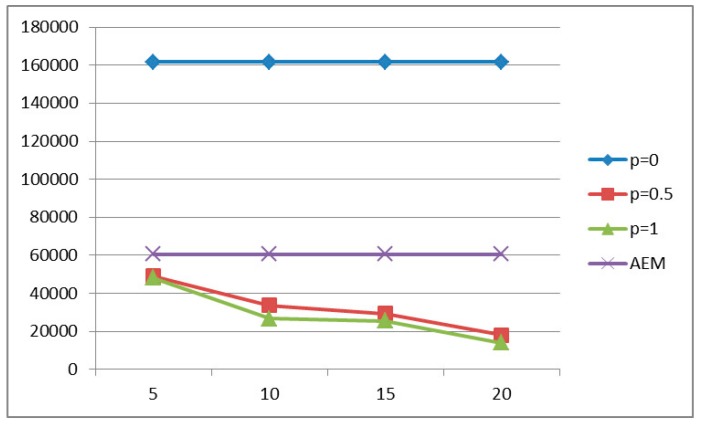
Energy Consumption.

As is illustrated in Figure 9, our approach succeeds in transmitting 90% of the generated packets, significantly outperforming all other alternatives, even when enforcing strict delay constraint and under high traffic conditions. Moreover, AEM achieves higher energy consumption efficiency compared to the other approaches since it consumes roughly the same amount of energy for transmitting significantly more packets, as observed in Figure 10.

## 6. Conclusions

Wireless sensor networks have been regarded as one of the promising and feasible solutions that may play an important role in the process of information sensing, collection and monitoring for various physical environments and, by extension, in several different smart city applications where services can be built upon, both for public and private use. Moreover, data aggregation techniques are considered an effective and viable paradigm for reducing the amount of traffic in sensor networks, thus increasing their lifetime and processing load. In this paper, an overall Optimization Framework for data gathering in resource-constrained sensor networks is introduced and evaluated. The proposed framework complements relevant work that can be found in the literature by identifying the optimal probability value for a node to aggregate packets as well as the optimal aggregation period that a node should wait, so as to minimize the overall energy consumption, while satisfying certain imposed delay constraints. Primal dual decomposition is employed to solve the corresponding optimization problem, while extensive simulation results demonstrate the efficiency of the proposed framework as well as its ability to adapt in challenging environments. The proposed algorithm, Aggregation-based Energy Management (AEM), which is based on the presented analysis, was compared with approaches that either do not perform aggregation or perform aggregation with constant probabilities and for fixed periods, under different traffic loads and application scenarios. As demonstrated by the performance evaluation process and respective simulation results, the proposed framework provides a robust paradigm, which achieves energy minimization, high probability of successful delivery and increased information gain at the cost of only a very small loss in the information accuracy. Finally, one of the key attributes of our framework is that is able to quickly adapt to changing environmental conditions, thus making it an ideal candidate for employment in harsh and dynamic environments and/or applications.
